# Multimodal Assessment and Characterization of Sicca Syndrome

**DOI:** 10.3389/fmed.2021.777599

**Published:** 2021-12-15

**Authors:** Emelie Kramer, Tabea Seeliger, Thomas Skripuletz, Vega Gödecke, Sonja Beider, Alexandra Jablonka, Torsten Witte, Diana Ernst

**Affiliations:** ^1^Department of Rheumatology and Immunology, Hannover Medical School, Hannover, Germany; ^2^Department of Neurology, Hannover Medical School, Hannover, Germany; ^3^Centre for Rare Diseases, Hannover Medical School, Hannover, Germany; ^4^Department of Nephrology, Hannover Medical School, Hannover, Germany

**Keywords:** sicca syndrome, Sjögren syndrome, lip biopsy, sicca symptoms, salivary gland ultrasonography (SGUS)

## Abstract

**Background:** Sicca syndrome represents a heterogeneous group of conditions, such as Sjögren syndrome, causing xerophthalmiaand xerostomia. This study characterizes in depth patients with Sicca syndrome and evaluates salivary gland ultrasound (SGUS).

**Methods:** Principal component analysis and hierarchical clustering of clinical parameters, such as ESSPRI, ESSDAI and laboratory data, were performed on all referrals for assessment of Sicca symptoms between October 2018 and March 2021. SGUS and labial gland biopsies were compared across groups.

**Results:** A total of 583 patients were assessed. Objective dryness was confirmed in 73% of the patients. Cluster analysis identified 3 groups with *post-hoc* analysis confirming distinct phenotypes: *Somatic Group* (283/583; 49%) with more frequent symptoms but limited objective dryness; *Dry Without Autoimmune Features* (DAF_neg_, 206/584; 35%), and *Dry With Autoimmune Features* (DAF_pos_, 94/584;16%). DAF_pos_ patients had highest autoantibody titers (anti-SSA(Ro) 240 vs. 3.6 vs. 3.8; *p* < 0.001), most extra-glandular manifestations (*p* < 0.001), and highest median SGUS Score (DAF_pos_: 8 [IQR 4–10] vs. SG: 2 [1–4] vs. DAF_neg_ 4 [2–5]; *p* < 0.001). No tangible correlation with primary Sjögren syndrome criteria was observed.

**Discussion:** SGUS score correlated with a subset of patients with Sjögren syndrome, identified in the DAF_pos_ cluster. This study highlights heterogeneity within sicca and, indeed, Sjögren syndrome, highlighting the need for further studies.

## Key Notes

- Patients exhibiting sicca symptoms, including those fulfilling criteria for primary Sjögren syndrome, are inherently heterogeneous, with inconsistent findings on salivary ultrasound.
- Novel clustering of patients with sicca symptoms incorporating principal component analysis of numerous relevant factors revealed 3 distinct phenotypes with distinct patterns of salivary gland involvement on ultrasound.
- Correlation between these clustered phenotypes and traditional definitions was limited, but suggested that clinically distinct subgroups among patients with Sjögren syndrome exist.
- Refinement of distinct disease entities within primary Sjögren syndrome appears feasible, and salivary gland ultrasound may assist in discrimination. Implications for future studies and, ultimately, tailored therapies require further evaluation.

## Background

Sicca syndrome can be considered an overarching term for symptomatic ocular (xerophthalmia) and oral dryness (xerostomia). Dryness is common, particularly with increasing age, affecting up to 30% of the population over 65 years (1). Current diagnostic approaches focus on determining if an autoimmune etiology exists while excluding drug side effects or manifestations of other systemic diseases that can either induce hyposecretion or lacrimal gland destruction (2). Sjögren syndrome encapsulates the autoimmune sicca syndrome and may be considered as primary Sjögren syndrome (pSS) when occurring in apparent isolation or secondary in the presence of another recognizable autoimmune condition. In practical terms, diagnosis centers on clinical history, objective measurements of xerophthalmia and xerostomia, and auto-antibody profiling. pSS requires evidence of autoimmune inflammation of salivary or lacrimal glands, as outlined in the joint American College of Rheumatology (ACR) and European League against Rheumatism (EULAR) classification criteria (3). These are deliberately broad, affording some heterogeneity in clinical features, but require either the presence of anti-SSA antibodies or evidence of lymphocytic sialadenitis on labial gland biopsy (LBx). Imaging is not required for the diagnosis of Sicca syndrome or the classification of pSS, but data for various modalities exist. Punctate calcification of parotid glands on computer tomography has demonstrated high diagnostic specificity, but utility is limited because of radiation exposure (4). Magnetic resonance imaging of the same glands has shown changes in both T1- and T2-weighted signal intensities (5). Salivary gland ultrasound (SGUS) has been purported as a low-cost and radiation-free alternative for many years, with hypoechoic lesions correlating with more severe disease in a variety of scoring systems (6–8). Furthermore, SGUS has proven to be an easily acquired diagnostic tool (9). Although specificity has been favorable, reported sensitivity has been moderate (10).

Given the inherent heterogeneity of such cohorts, such as the heterogeneous group of patients with pSS and varying manifestations, this finding is perhaps unsurprising (11, 12). The aim of this study is to independently assess the role of SGUS in a large, unselected Sicca syndrome cohort that has undergone extended-criteria phenotypic clustering.

## Methods

All patients referred for rheumatological assessment of suspected Sicca syndrome at our Institution between October 2018 and March 2021 were prospectively included. Structured clinical data, assessing symptoms, and ESSPRI and ESSDAI scores were collected. All the patients were tested for antinuclear and anti-SSA(Ro)/anti-SSB(La) antibodies, rheumatoid factor along with differential blood count, and standard biochemistry indices. Xerophthalmia was assessed by Schirmer test and xerostomia by Saxon test in all the patients, and < 3.5 g in 2 min (stimulated saliva flow) and < 5 mm in 5 min (lacrimal flow) were considered as reduced. LBx was performed in the patients with suspected pSS as indicated and graded according to Chisholm Mason Score, with grade ≥ 3 being considered diagnostic (13). SGUS consisted of bilateral assessment of both parotid and submandibular glands and was performed by 2 blinded sonographers experienced in the procedure. Image interpretation adhered to criteria defined by a score from 0 to 3 depending on homogeneity, and both cumulative totals and DeVita scores were considered in the analysis (6, 14). All scans were independently re-scored by the non-performing sonographer, and a consensus score derived from both scores was used in the analysis. In case of disagreement, the images were reviewed, and the lower scores were employed. All the participants provided informed written consent prior to inclusion, and the study was approved by the Institutional Review Board at Hannover Medical School (8179_BO_S_2018).

With the exception of SGUS and LBx scores, all collected continuous variables were included in a principal component analysis. Missing values were estimated by multiple imputation, and the calculated dimensions were evaluated by hierarchical clustering. The sensitivity and specificity of both SGUS and LBx within the identified clusters were then evaluated. All the statistical analyses were performed using R v4.0.3 (R Foundation, Vienna, Austria). Multiple imputations were performed using the missMDA package, and the FactoMineR package for principal component analysis and hierarchical clustering. Graphs were created using ggplot2, scatterplot3d, and rgl packages where appropriate. In cases where three or more groups were compared, Kruskal Wallis test was performed for categorical variables and ANOVA tests for comparing quantitative variables, where appropriate. The Mann-Whitney test was performed for two variables.

## Results

**Figure 1:**
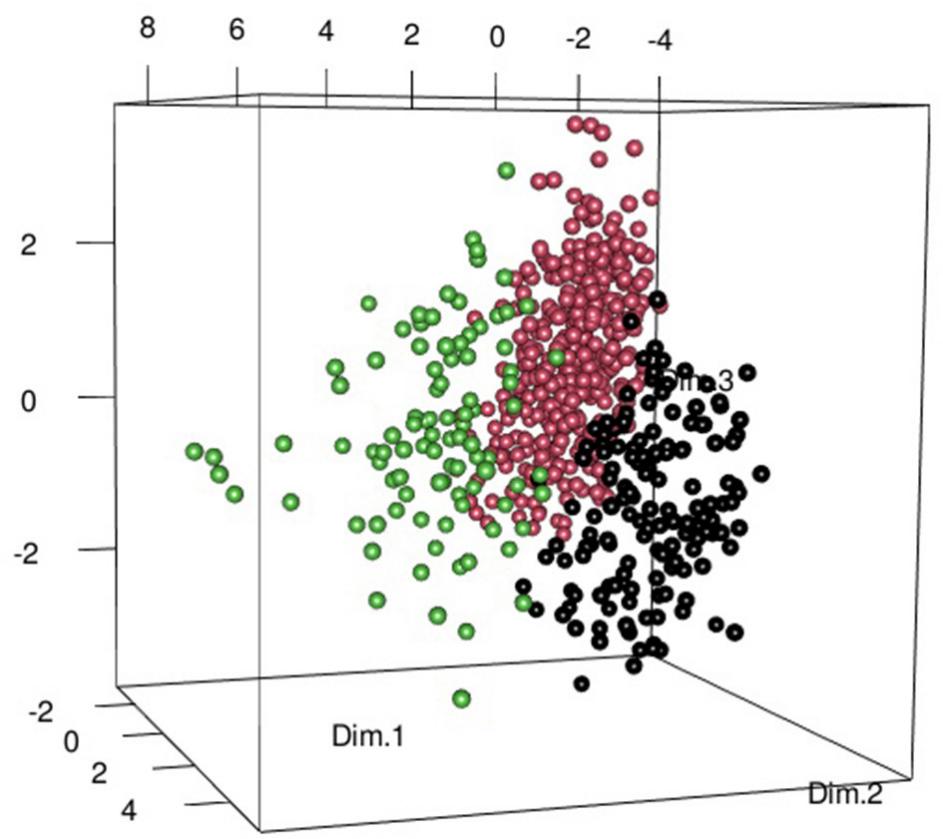
Three-dimensional (3D) scatter plot composed of the first 3 dimensions of the principal component analysis, identified as providing greatest inertia gain on hierarchical clustering. The Somatic Group (SG) is represented by the black points. Patients in the Dryness without autoimmune features (DAF_neg_) group are represented by pink points and Dryness with autoimmune features (DAF_pos_) patients with green dots. This Figure was produced using the scatterplot3d and rgl packages.

A total of 583 patients were included; the majority of whom were female (462/583, 79%). Median age at symptom onset was 56 [interquartile range (IQR) 49.5–68] years. After subjective dryness, the most common symptoms were arthralgia (*n* = 401, 69%) and myalgia (*n* = 324, 56%). Objective dryness, defined as positive Schirmer and/or Saxon test, was observed in 425 (73%) of the patients. None of those included had previously undergone radiotherapy or were receiving tricyclic antidepressants at the time of inclusion. Applying the ACR/EULAR pSS criteria across the cohort, in total, 231 (40%) fulfilled the classification criteria and 85 (15%) possessed none of required features (Supplementary Figure 1). A comprehensive summary of the entire cohort is included in the supplementary data. Following principal component analysis, hierarchical clustering identified three clearly demarcated groups (Figure 1), which were then phenotypically characterized in detail (Table 1).

**Table 1:** Comparing and contrasting the clinical demographics and attributes of the entire cohort subdivided into the three groups identified through principal component analysis and subsequent hierarchical clustering.

	**Somatic**		**DAF**_**neg**_		**DAF**_**pos**_		***p-*value**
N (%)	283	(49)	206	(35)	94	(16)	
Female, *n* (%)	239	(84)	142	(69)	81	(86%)	<0.001
Age at Onset, years	47.3	[36.6–55.9]	60.2	[51.1–67.3]	50.1	[35.5–59.4]	<0.001
BMI, kgm^−2^	26.1	[23.0–31.0]	24.7	[21.7–27.7]	24.6	[22.4–28.0]	0.003
Smoker, *n* (%)	50	(18)	15	(7)	4	(4)	0.06
**ESSPRI Scores**							
-Dryness	6	[3–7]	2	[1–3]	4	[2–4]	<0.001
-Limb Pain	7	[5–8]	5	[2–6]	6	[4–8]	
-Fatigue	8	[6–9]	3	[2–5]	5	[3–8]	
**Reported Symptoms**							
Raynaud, *n* (%)	86	(30)	51	(25)	44	(47)	0.006
Arthralgia, *n* (%)	222	(78)	118	(57)	61	(65)	0.002
Myalgia, *n* (%)	197	(70)	87	(42)	40	(43)	0.003
Stiffness, *n* (%)	98	(35)	37	(18)	23	(25)	0.001
Parotitis, *n* (%)	62	(22)	24	(12)	33	(35)	0.001
Sand corn^*^, *n* (%)	168	(59)	62	(30)	44	(47)	0.001
Ocular Inflammation, *n* (%)	120	(42)	45	(22)	26	(27)	0.005
**ESSDAI**							
-Score	5	[2–12]	5	[0–11]	11	[4–17]	<0.001
ESSDAI Constitutional, *n* (%)							0.02
-None	194	(69)	173	(84)	74	(79)	
-Low	79	(28)	26	(13)	16	(17)	
-Moderate	10	(3)	7	(3)	4	(4)	
ESSDAI Lymphadenopathy, *n* (%)							0.09
-None	232	(82)	182	(88)	88	(94)	
-Low	50	(18)	24	(12)	4	(4)	
-Moderate	1	(<1)	-	-	1	(1)	
-High	-	-	-	-	1	(1)	
ESSDAI Glandular Involvement, *n* (%)							0.21
-None	263	(93)	197	(96)	85	(90)	
-Low	19	(7)	8	(4)	8	(9)	
-Moderate	1	(<1)	1	(<1)	1	(1)	
ESSDAI Articular Involvement, *n* (%)							0.002
-None	241	(85)	199	(97)	73	(78)	
-Low	37	(13)	3	(1)	14	(15)	
-Moderate	4	(1)	4	(2)	7	(7)	
-High	1	(<1)	-	-	-	-	
ESSDAI Cutaneous Involvement, *n* (%)							0.16
-None	262	(93)	195	(95)	83	(89)	
-Low	14	(5)	6	(3)	5	(5)	
-Moderate	7	(2)	4	(2)	6	(6)	
-High	-	-	1	(<1)	-	-	
ESSDAI Pulmonary Involvement, *n* (%)							0.03
-None	251	(89)	177	(86)	73	(78)	
-Low	16	(6)	8	(4)	4	(4)	
-Moderate	12	(4)	11	(5)	11	(12)	
-High	4	(1)	10	(5)	6	(6)	
ESSDAI Renal Involvement, *n* (%)							0.26
-None	279	(98)	206	(100)	89	(95)	
-Low	2	(1)	-	-	3	(3)	
-Moderate	-	-	-	-	2	(2)	
-High	2	(1)	-	-	-	-	
ESSDAI Muscular Involvement, *n* (%)							0.38
-None	272	(96)	197	(96)	87	(93)	
-Low	5	(2)	6	(3)	1	(1)	
-Moderate	4	(1)	3	(1)	5	(5)	
-High	2	(<1)	-	-	1	(1)	
ESSDAI Peripheral Nerve Involvement, *n* (%)							0.77
-None	206	(73)	154	(75)	65	(69)	
-Low	35	(12)	15	(7)	7	(7)	
-Moderate	32	(11)	22	(11)	17	(18)	
-High	10	(4)	15	(7)	5	(5)	
ESSDAI Central Nerve Involvement, *n* (%)							0.13
None	263	(93)	200	(97)	88	(94)	
Moderate	9	(3)	1	(<1)	2	(2)	
High	11	(4)	5	(2)	4	(4)	
ESSDAI Hematological Involvement*, n* (%)							0.012
-None	223	(79)	163	(79)	51	(54)	
-Low	51	(18)	36	(18)	32	(34)	
-Moderate	8	(3)	6	(3)	11	(12)	
-High	1	(<1)	1	(<1)	-	-	
ESSDAI Biological Involvement, *n* (%)							<0.001
-None	237	(84)	167	(81)	49	(52)	
-Low	36	(13)	33	(16)	21	(22)	
-Moderate	10	(3)	6	(3)	24	(26)	
**Antibody Titres**							
-ANA ≥ 1:160	178	(63)	152	(74)	44	(47)	<0.001
-RhF U/ml	10.0	[10.0–10.9]	10.0	[10.0–11.3]	23.3	[11.7–71.0]	<0.001
-Alpha-Fodrin U/ml	9	[5–22]	9	[6–19]	12	[6–25]	0.05
-anti-SSA(Ro) U/ml	3.6	[0.3–101.3]	3.8	[0.3–102.3]	240.0	[192.8–240.0]	<0.001
-anti-SSB(La) U/ml	0.4	[0.3–3.4]	0.3	[0.3–1.9]	73.1	[3.8–312.5]	<0.001
**Measurable Dryness**							
Saxon, g	3.5	[2.4–4.9]	4.2	[3.3–5.3]	2.3	[0.6–3.7]	<0.001
Schirmer, mm	7.0	[2.0–17.9]	3.0	[0.5–12.0]	2.5	[0.0–7.1]	<0.001
**Labial Gland Biopsy**, ***n*** **(%)**							
-Biopsy performed	150	(53)	120	(58)	18	(19)	
-Chisholm grade ≥3	66	(44)	64	(53)	9	(50)	
-Median Score	2	[1–3]	3	[2–3]	3	[3–4]	
**Salivary Gland Ultrasound**, ***n*** **(%)**							
-SGUS = 0	39	(14)	39	(19)	1	(1)	
-SGUS ≥ 6	73	(26)	55	(27)	38	(41)	
SGUS Score	2	[1–4]	4	[2–5]	8	[4–10]	<0.001

*The results are shown as mean and interquartile range unless stated otherwise*.

* *Foreign body or grain of sand feeling in the eye*.

Almost half (283/583; 49%) of the patients largely lacked objective abnormalities. Interestingly, these patients reported most subjective dryness, as well as highest pain and fatigue scores, and were referred to as the somatic group (SG). Just over a third of the patients (206/583; 35%) exhibited objective dryness mainly in the absence of autoimmune features (DAF_neg_). Xerophthalmia was particularly prevalent, with patients being older and less likely to be female. Anti-SSA(Ro) antibodies were generally negative or of low titers in these patients. Ninety-four (16%) patients displayed objective dryness with autoimmune features (DAF_pos_). These patients had the most severe xerophthalmia and xerostomia, most prevalent and markedly higher anti-SSA(Ro) antibody titers, and tended to be younger at the time of disease onset.

LBx was performed on only 288 (49%) of the patients. Given the high probability of selection bias, no statistical analysis was performed within the subgroups. A Chisholm grade ≥ 3 was observed in 139 (48%) of the biopsies performed. Of these, 101 (73%) were associated with a pathologic Saxon and/or Schirmer test, whereas only 34 (24%) corroborated a positive anti-SSA(Ro) antibody. The latter point may be at least partially explained by lower referrals for biopsy in anti-SSA(Ro)-positive patients. This can be seen within the clustered phenotypes, where only 19% of the DAF_pos_ patients underwent biopsy, compared to the 58% of DAF_neg_ and 53% of the SG. Although histologic grading tended to be slightly higher among the DAF_pos_ patients, the proportion of biopsies considered positive was similar across all the groups.

**Figure 2:**
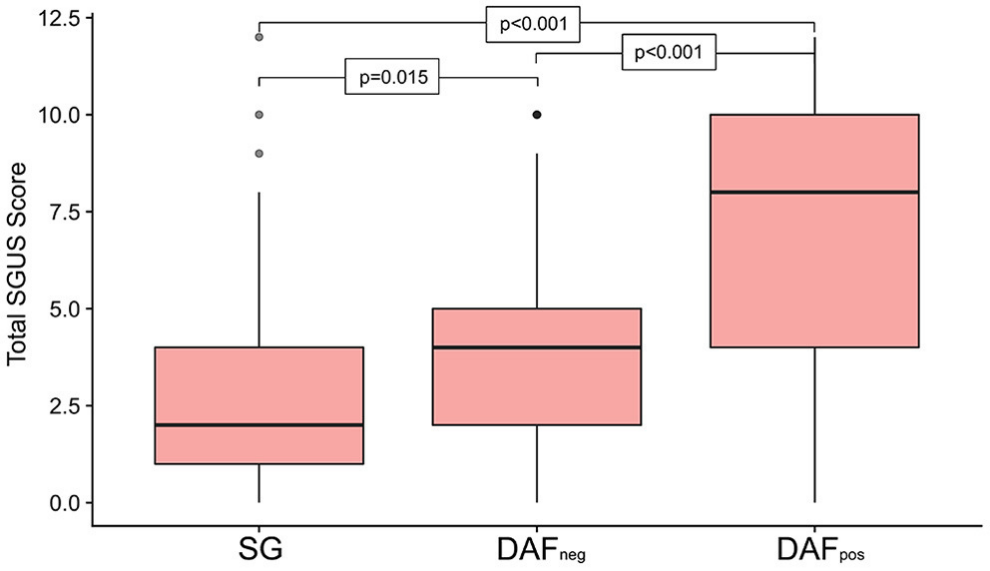
Boxplot summarizing the cumulative ultrasound score for all four sites investigated, subdivided by phenotype group. Significant differences were seen across all the 3 groups, with patients in the Dryness with autoimmune features (DAF_pos_) group returning the highest scores. Upper and lower box margins represent the interquartile range, with the central dark horizontal line representing the median score. Key: DAF_neg_, dryness without autoimmune features; DAF_pos_, dryness with autoimmune features; SG, somatic group.

Salivary gland ultrasound (SGUS) was performed on all the patients, with cumulative scores ranging from 0 to 12 and a median score of 4 [IQR 2–6] across the entire cohort. Sixty patients (10%) had no detectable SGUS abnormality, and all but 2 were from the DAF_neg_ group (Supplementary Figure 2). Only 7 (7%) of the DAF_pos_ patients exhibited an SGUS total of < 5 (Supplementary Figure 2). Overall, the median SGUS score was lowest in the SGat 2 [IQR 1–4], with the DAF_neg_ group returning a median score of 4 [IQR 2–5]. Although the DAF_pos_ group scored much higher with a median of 8 [IQR 4–10], differences among all the groups proved highly significant (Figure 2). Furthermore, distinct patterns of glandular involvement were observed, with parotid involvement being almost exclusively occurring in the DAF_pos_ group (Supplementary Figure 3). With regard to fulfillment of pSS criteria in somatic and DAF_neg_ patients, a much higher proportion was observed in those with SGUS ≥ 6 vs. SGUS < 6 (84 vs. 55%). A full description of the relationship between pSS criteria and cumulative SGUS score is included in the supplementary data (Supplementary Figure 2).

## Discussion

This study evaluated the utility of SGUS in unselected patients referred for evaluation of Sjögren syndrome, and explored the relationship between Sicca syndrome and Sjögren syndrome beyond the ACR/EULAR criteria. Reported dryness proved an unreliable feature, with over a quarter of the patients lacking measurable xerophthalmia and xerostomia. Forty percent of the patients fulfilled the pSS criteria. Close scrutiny revealed significant heterogeneity among the patients, and indeed SGUS could not reliably characterize patients in this group. Given these observations and cohort size, we instead considered all available data to develop a more granular, inclusive characterization of sicca syndrome, better reflecting everyday clinical decision-making. This identified three distinct groups that were compatible with everyday clinical experiences. Clearly the DAF_pos_ group is small and represents a subgroup of more active pSS, given the higher ESSDAI scores and that all but one patient fulfilled the pSS criteria. However, sizable minorities in both the DAF_neg_ and SG groups also fulfilled the pSS criteria. Further interpretation comparing the calculated phenotypes and pSS should be discouraged, given the limited number of LBx performed. While ethically difficult to justify without a clear clinical indication, it is likely that fulfillment of the pSS criteria is underreported. Interestingly, positivity rates of labial gland biopsies were almost identical across all the groups.

The most significant finding was the exceptional correlation between the SGUS and DAF_pos_ patients, particularly with regard to parotid gland abnormalities. There has been some debate about the utility of SGUS as an additional criterion for pSS (15, 16), and our data would support its utility in identifying the most active patients. Indirectly, this corroborates a recent pSS study in which 29% of patients with pSS and low SGUS scores were considered to have milder disease (17).

Despite the large sample size, the retrospective nature of this analysis and the limited availability of LBx limit the interpretation of outcomes. Although both sonographers were blinded during data collection, further limitations arise from using only two observers. No adjustments were made for symptom duration before imaging. This would require serial rescanning and longitudinal analysis, which are beyond the scope of this study.

In conclusion, sicca syndrome comprises a heterogeneous group of conditions in which Sjögren syndrome remains an awkward fit. Performing novel statistical analysis on a broader range of parameters, three distinct phenotypes were identified. Parotid gland involvement in SGUS occurred almost exclusively in patients with autoimmune features. More research is needed to explore this relationship over time and further refine models evaluating sicca syndrome, and in turn assist treatment study design with a view to tailored therapy strategies.

## Data Availability Statement

The raw data supporting the conclusions of this article will be made available by the authors, without undue reservation.

## Ethics Statement

The studies involving human participants were reviewed and approved by Institutional Review Board at Hannover Medical School (8179_BO_S_2018). The patients/participants provided their written informed consent to participate in this study.

## Author Contributions

EK: data collection, writing of the manuscript, database work, and patient flow. TSe: data collection and critical review. TSk, SB, AJ, and VG: critical review. TW: critical review and study design. DE: study design, organization, analyses, and writing of the manuscript. All authors contributed to the article and approved the submitted version.

## Conflict of Interest

This study was financially supported by Novartis and the Else-Kröner Charity. The funder was not involved in the study design, collection, analysis, interpretation of data, the writing of this article or the decision to submit it for publication. The authors declare that the research was conducted in the absence of any commercial or financial relationships that could be construed as a potential conflict of interest.

## Publisher's Note

All claims expressed in this article are solely those of the authors and do not necessarily represent those of their affiliated organizations, or those of the publisher, the editors and the reviewers. Any product that may be evaluated in this article, or claim that may be made by its manufacturer, is not guaranteed or endorsed by the publisher.
