# Supplementary material for: Multimodal Assessment and Characterization of Sicca Syndrome
Source: Front Med (Lausanne). 2021 Dec 15;8:777599. doi: 10.3389/fmed.2021.777599 (PMC8714883; doi:10.3389/fmed.2021.777599)
Supplement: Supplementary file 1 [file Data_Sheet_1.docx]

Multimodal Assessment & Characterization of Sicca Syndrome

Emelie Kramer^1^, Tabea Seeliger^2^, Thomas Skripuletz^2^, Vera Gödecke^3,4^, Sonja Beider^1^, Alexandra Jablonka^1^, Torsten Witte^1^, Diana Ernst^1^

| ^1^ | Department of Rheumatology,and Immunology Medical School Hannover, Hannover, Germany |
| --- | --- |
| ^2^ | Department of Neurology, Medical School Hannover, Hannover, Germany |
| ^3^ | Centre for rare diseases, Medical School Hannover, Hannover, Germany |
| ^4^ | Department of Nephrology, Medical School Hannover, Hannover, Germany |

Supplementary Data: Multimodal Assessment & Characterization of Sicca Syndrome

|  | 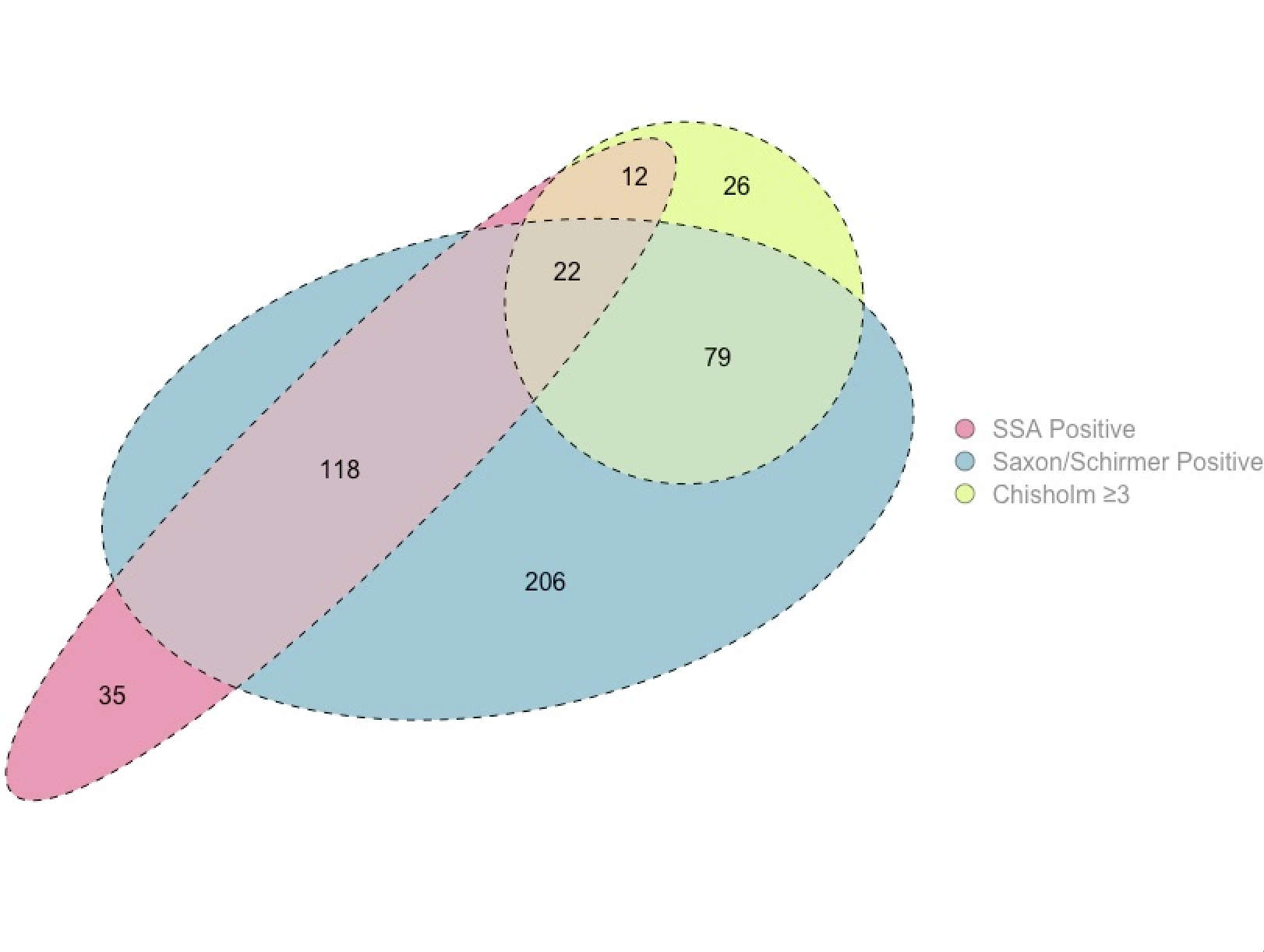 |  |
| --- | --- | --- |
| Fig. S1 Euler Diagram illustrating the distribution of individual ACR/EULAR criteria for primary Sjogren syndrome across the entire cohort of patients with sicca symptoms. It is important to emphasize that labial gland biopsies were only performed when indicated (N=288), and are most likely under-represented. | | |
|  |  |  |
| \|  \| 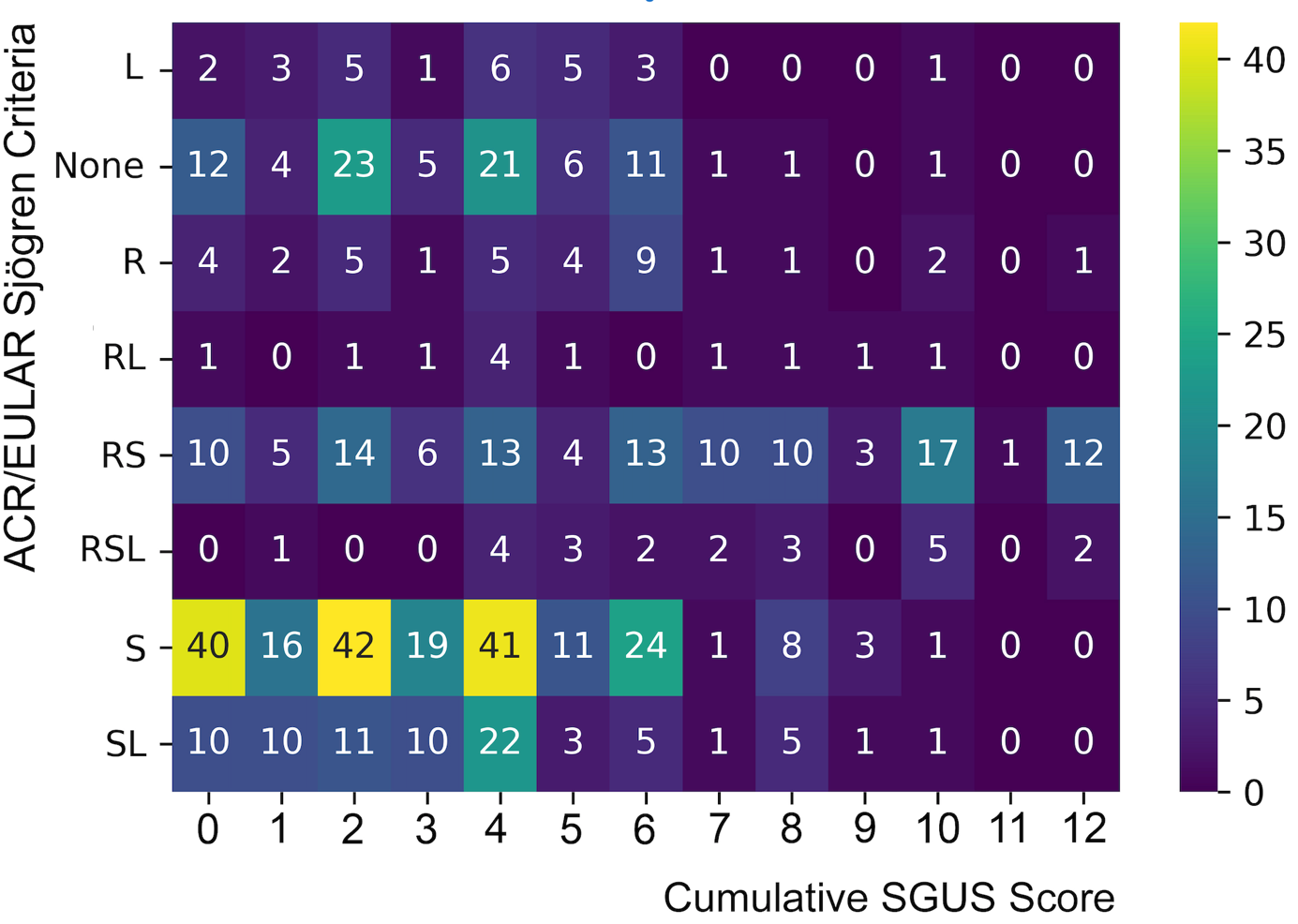 \|  \| \| --- \| --- \| --- \| \| Fig. S2 – Heatmap comparing cumulative ultrasound scores with the prevalence of current American College of Rheumatology / European League Against Rheumatism (ACR/EULAR) criteria for diagnosing Sjögren Syndrome. Numbers in the map represent the number of patients with both feature sets. Key: L – Chisholm Lip Biopsy Score ≥3; R – Anti-SSA (Ro) antibody positive; S – Saxon and/or Schirmer test achieving positivity. \| \| \| | | |
|  | 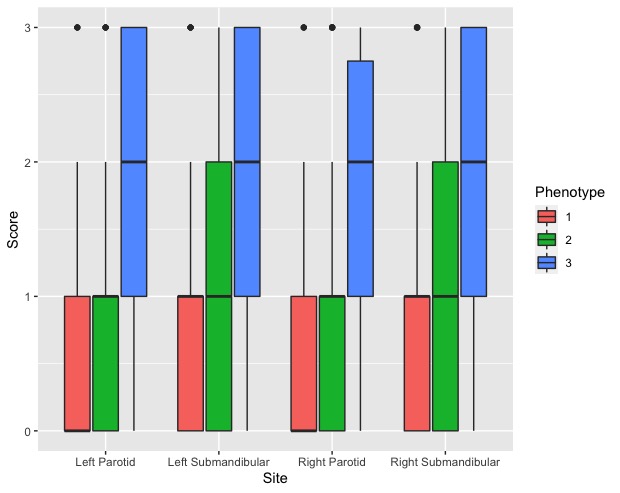 |  |
| Fig. S3 – Boxplot depicting recorded salivary gland ultrasound score (SGUS) at each of the four sites tested. *Somatic Group* (red boxes) consistently returned the lowest scores at all sites. *Dry without autoimmune features* (DAF_neg_) patients (green boxes) returned higher submandibular scores than parotid scores, whereas *Dry with autoimmune features* (DAF_pos_) patients (blue boxes) returned similar, significantly higher scores at all sites. | | |
